# GABA_A_ Receptors in Normal Development and Seizures: Friends or Foes? 

**DOI:** 10.2174/157015908783769653

**Published:** 2008-03

**Authors:** Aristea S Galanopoulou

**Affiliations:** Albert Einstein College of Medicine, Saul R Korey Department of Neurology & Dominick P Purpura, Department of Neuroscience, Bronx NY, USA

**Keywords:** GABA, chloride, brain, development, seizure, hippocampus, expression, physiology.

## Abstract

GABA_A_ receptors have an age-adapted function in the brain. During early development, they mediate excitatory effects resulting in activation of calcium sensitive signaling processes that are important for the differentiation of the brain. In more mature stages of development and in adults, GABA_A_ receptors transmit inhibitory signals. The maturation of GABA_A_ signaling follows sex-specific patterns, which appear to also be important for the sexual differentiation of the brain.

The inhibitory effects of GABA_A_ receptor activation have been widely exploited in the treatment of conditions where neuronal silencing is necessary. For instance, drugs that target GABA_A_ receptors are the mainstay of treatment of seizures. Recent evidence suggests however that the physiology and function of GABA_A_ receptors changes in the brain of a subject that has epilepsy or status epilepticus.

This review will summarize the physiology of and the developmental factors regulating the signaling and function of GABA_A_ receptors; how these may change in the brain that has experienced prior seizures; what are the implications for the age and sex specific treatment of seizures and status epilepticus. Finally, the implications of these changes for the treatment of certain forms of medically refractory epilepsies and status epilepticus will be discussed.

## INTRODUCTION 

GABA (γ-aminobutyric acid) was discovered in the brain in 1950 [11, 265, 309] and has since been established as the primary inhibitory neurotransmitter in the brain [48, 155]. Paradoxically, GABA is derived from the prototypical excitatory neurotransmitter glutamate [265], declaring even from its early production steps its role as a shunt of excitatory inputs, in a network where the balance between excitation and inhibition is very sensitive. GABA can bind to metabotropic GABA_B_ receptors [133, 142, 143, 157, 221, 324] or to iono-tropic GABA_A_ or GABA_C_ receptors. Activation of postsynaptic GABA_B_ receptors increases membrane conductance to K+ leading to prolonged neuronal hyperpolarization. Presynaptic GABA_B_ receptor activation reduces Ca++ conductance and neurotransmitter release. Ionotropic GABA_A_ or GABA_C_ receptors are in turn permeable to chloride and bicarbonate ions [69]. Classically, activation of GABA_A_ or GABAC receptors allows the influx of Cl-, following its electrochemical gradient, resulting in neuronal hyperpolarization. However, early during development, ionotropic GABA receptors mediate depolarizing currents, which activate calcium sensitive signaling processes that are vital for neuronal differentiation and brain development. The importance of GABA-mediated inhibition in normal brain function and diseases stemming from imbalance of excitation and inhibition is well accepted, thanks to our increasing knowledge of brain physiology, pharmacological advances and the progress of genetics. This has rendered the GABA pathway a popular target of pharmacological interventions when excessive brain excitation needs to be averted. However, the changing role of GABA during development and under certain pathological conditions has triggered a line of research re-evaluating the acute and long term effects of GABAergic drugs in the naïve developing brain or the brain that has experienced insults such as seizures. The current review will discuss the current state of knowledge about the dual actions of GABA, specifically as they pertain to GABA_A_ receptor signaling, in the context of normal brain development or of a brain that has experienced seizures.

## GABA_A_ RECEPTORS IN NORMAL BRAIN FUNCTION AND DEVELOPMENT

### GABA_A_ Receptors: Structure and Pharmacology

GABA_A_ receptors are pentameric channels composed of different combinations of subunits, with distinct pharmacological, localizing or kinetic properties [17]. In mammalians, 16 GABA_A_ receptor subunits are known (α1-α6, β1-β3, γ1-γ3, δ, ε, θ, and π) which form bicuculline-sensitive, ligand-gated ion channel complexes [7, 24, 94-96, 100, 108, 110, 113, 132, 150, 168, 174, 175, 186, 194, 208, 233, 269, 276, 277, 282, 289, 329, 330, 332, 335, 337-339]. Alternate splicing offers additional diversity [18, 55, 152, 160, 248, 325]. The inclusion of a ρ subunit (ρ1 – ρ3) distinguishes the bicuculline-insensitive GABA_C_ receptor family [27, 36, 50, 67, 91, 176, 228, 229, 322, 344]. Two more subunits (β4 and γ4) have been identified in chicken [101, 161]. The obligatory components of a functional GABA_A_ receptor complex are the α and β subunits, typically 2 from each type. Channels formed only as a combination of α and β subunits can be functional [64, 250], but most frequently, a γ or δ subunit is also included. α_x_β_y_γ complexes are usually synaptically located GABA_a_ receptors, mediating phasic inhibition [206, 208], although similar extrasynaptic complexes have also been reported [46, 68, 225, 226]. They are activated upon the spontaneous or triggered vesicular release of GABA and are therefore responsible for phasic GABA_A_ receptor inhibitory postsynaptic currents (IPSCs). Alternatively, a δ, θ, π, or ε subunit may be included in the receptor complex. The presence of a δ subunit typically directs the GABA_A_ receptor complex to extrasynaptic locations, where GABA_A _receptors are tonically activated by ambient GABA [226]. Ambient GABA may rise in cases of excessive synaptic GABA release and spillover, as occurs in seizures, or through pharmacological blockade of GABA re-uptake mechanisms. Less frequently extrasynaptic receptors are composed of αβ or α5β 3γ2 or a1β2γ2 or α3β3γ2, if highly expressed [21, 131, 211, 226, 296]. 

The combination of different subunits determines the pharmacological characteristics, kinetics, and subcellular localization of the GABA_A_ receptors (reviewed in [208]). For example, zolpidem is an α1-selective agonist [207, 271]; 4,5,6,7-tetrahydroisoxazolo-[5,4-c]pyridin-3-ol (THIP or gaboxadol) has highest affinity for δ-subunit containing receptors [2, 298]. Their modulatory domains include binding sites for benzodiazepines (BZ site), GABA, barbiturates, nonbarbiturate anesthetics and ethanol, neurosteroids, picrotoxin, penicillin, and zinc. Among these, the BZ site is best characterized. Typical agonists at the BZ site are diazepam and lorazepam, whereas typical antagonist is flumazenil. Each receptor complex may have up to 2 BZ sites, each at the interface of an α and γ subunit, and up to 2 GABA sites, between an α and β subunit. Depending upon the subunit composition, BZ sites may have different ligand affinities, highest at type I sites (preferably α1-containing), intermediate at type II_M_ (preferably α2- or α3- containing) and low at type II_L_ (α5-containing) [197]. Among the γ subunits, γ2 is preferred for high BZ affinity [208]. Benzodiazepines have high affinity for most α and γ subunit containing receptors with exception of α4 and α6, and very low affinity for δ-containing receptors [207, 208]). Affinity to barbiturates affinity is determined by the β-subunit [99] and the α-subunit present [307], whereas ε-containing receptors are insensitive to barbiturates and other anaesthetic agents [54]. Neurosteroids typically act upon a δ-containing receptor complex, although α1β1γ2 or α3β1γ2 complexes may be responsive to their effects [21, 208]. GABA_A_ receptor agonists can act as GABA-modulatory drugs, altering the effects of GABA binding, such as benzodiazepines, or GABAmimetic, which directly activate the receptors in the absence of GABA, such as muscimol, barbiturates or neurosteroids at high doses [185, 187]. 

Apart from GABA, several naturally occurring GABA_A_ receptor-acting compounds have been identified. The benzodiazepine-like compounds diazepam and N-desmethyldiaze-pam have been detected in rat brain and adrenals [326], bovine cerebral cortex and milk [195], human milk [243] and have been localized into synaptic vesicles with immunocytochemical assays [195]. It is yet unclear whether these can be synthesized in these organisms *in vivo* or whether they are ingested from food products, such as wheat (diazepam [328]) or potato (lormetazepam, desmethyldiazepam, delorazepam, lorazepam, delormetazepam [272, 327]). *In vivo* biosynthetic pathways for N-desmethyldiazepam have been described in the fungus* Penicillium verrucosum* [31, 32]. In rat brain, active benzodiazepines can be generated *in vivo* from tryptophan [196] or during *in vitro* incubation [245]. *Acinetobacter lwoffii*, a bacterium of the intestinal or skin flora can produce inactive precursors of BZ-like molecules [341]. Pathological accumulation of their active benzodiazepine derivatives has been described in cases of hepatic failure and may contribute to hepatic encephalopathy. 

### Developmental Changes in GABA_A_ Receptor Structure and Pharmacology

Most studies describing developmental changes in GABA_A_ergic signaling have been done in rats. To better understand how might these reflect changes in humans, it is generally thought that brain development in a postnatal day 8-10 (PN8-10) rat is almost equivalent to a newborn human baby. The infantile stage in rats spans from PN7-21 and is followed by the juvenile stage. Puberty onset in rats occurs at approximately P32-37, whereas adulthood is reached at 2 months [230, 342, 343]. GABA is present in the embryonic neural system from the very early days [105, 162]. In the embryonic rat neocortex, GABA is detected diffusely as early as embryonic day 10 (E10) but after E14 its presence is limited to the subplate, cortical plate, marginal and intermediate zones [105]. In parallel, GABA_A_ receptors are expressed, even before the establishment of GABAergic synapses, to permit the autocrine and paracrine actions of GABA on brain development [164, 183, 278]. Regional differences in subunit expression have been reported in rats, with α4, β1, γ1 detected in the premigratory neuroblasts of the ventricular zone [164, 183] and α2, α3, β3, γ2  at the cortical or subcortical plate [164, 183, 190]. The spatiotemporal developmental patterns of GABA / GABA_A_ receptor expression are thought to be important in the orchestration of the normal GABA-related regulation of proliferation and migration or neural and glial progenitors [105]. The high levels of GABA in the early stages of development promote the proliferation of ventricular zone progenitors [105], whereas the subsequent decline and restriction of GABA_A_ergic influence within the outer neocortical layers inhibits proliferation [8, 105, 177], enhances migration [20], and may therefore permit further neuronal differentiation. GABA_A_ergic signaling is also important for neuronal survival at this stage [128]. In further support of the importance of GABA_A_ergic signaling for brain development, *in utero* exposure to GABA_A_ receptor inhibitors decreases the number of parvalbumin-immuno-reactive GABAergic neurons in the striatum, by impairing the survival or differentiation of these neurons [182]. Moreover, focal application of GABA_A_ergic agonists in the cortex of newborn rats may induce abnormal migration and heterotopias [107].

**Fig. (1).**
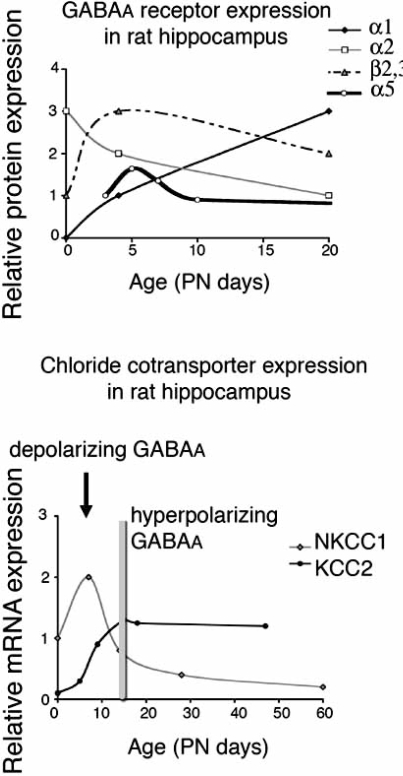
**Developmental changes in selected GABA_A_ receptor subunits, chloride cotransporters (KCC2 and NKCC1) and GABA_A_ receptor physiology in rat hippocampus**.*Upper panel:* A developmental decrease in α2 and α5 in parallel with an increase in a1 has been described in rat hippocampus. Age dependent changes in other subunits, such as β2,3 has also been reported. Results are from studies [34, 74, 89, 120, 140, 253, 204]. The scale is arbitrary and intends to depict relative changes in expression of a given subunit across ages, and not the relative abundance of one subunit *vs* another. *Lower panel*: A switch from an NKCC1-dominant to a KCC2-dominant state occurs in infantile rat hippocampus and has been implicated in the functional switch of GABA_A_ receptor signaling from depolarizing to hyperpolarizing. Results are compiled from studies in male rats or rats of undetermined sex [247, 261]. The vertical bar indicates the age when hyperpolarizing GABA_A_ receptor signaling occurs.

Age-related, species, and region-specific changes, gradual or transient, continue through postnatal development, adulthood and ageing for GABA_A_ receptor subunits like α1, α2, α3, α4, α5, γ1, γ2 [138, 171, 214, 255, 260, 340]. Fritschy *et al*. have proposed that during the early postnatal life, a gradual parallel decrease in α2 / α3 and increase in α1 expression occurs in rat brain [74, 120] (Fig. **1**). Similar developmental switch from α2 / α3 to  α1 subunit predominance has been observed in mouse superior colliculus [111] and visual cortex [37, 109]. Functionally, the postnatal increase in α1 has been linked to increased sensitivity to neurosteroids [214], zolpidem [111] and benzodiazepines [140], and acquisition of mature type postsynaptic IPSCs with shorter duration [29]. The latter may be important for a brain that learns to respond appropriately to novel patterns of neuronal activation. Using α1 knockout mice, Bosman *et al*. have elegantly shown that lack of α1 subunits leads to preservation of juvenile, long duration IPSCs and impairs spatiotemporal excitation patterns to local high frequency stimulation in the visual cortex [28, 29]. In the dentate granule cells of the rat hippocampus, the developmental switch from α5 to α1, α4, and γ2 subunits correlates with decreasing sensitivity to zinc and increase in the affinity for benzodiazepines [34, 140]. Sensitivity to zinc is important in the functional regulation of GABA_A_ergic transmission, particularly in immature neurons. Large amounts of zinc can be stored in synaptic vesicles of nerve terminals, as in the hippocampal mossy fibers of the immature hippocampus. Stimulation-dependent zinc release in this system may therefore be useful to keep under control the excessive depolarizing effects of GABA, in a subunit-specific pattern [16, 53, 166, 285, 331]. This may be less important in adult neurons, which lose their sensitivity to zinc, as GABA_A_ receptor mediated inhibition is more efficient.

There is though regional specificity of the evolution of these changes [56]. Sex differences in GABA_A_ receptor subunit expression further increase the diversity. These include increased expression of α1 subunit in the female substantia nigra of infantile and juvenile rats [255] and increased γ1 expression in the male rat juvenile medial preoptic area [219]. At the cellular level, GABA_A_ receptor trafficking also evolves. Early in development and before synaptic integration occurs, receptor complexes can be diffusely expressed at the cell membrane and can be tonically activated in the presence of GABA [61, 172, 177, 236, 311]. As the establishment and differentiation of GABAergic synapses begins, they initially occupy both extrasynaptic and synaptic sites; finally targeting and clustering at synaptic sites and dendritic processes increases with maturation and spontaneous IPSCs can be detected [1, 236, 253]. 

The temporal, regional, sex, and species specific variability in the expression of these subunits in the brain emphasizes that generalization across brain regions, species, genders, and ages is not possible, but one needs to specifically study each structure, age, and condition independently. To further complicate these studies, handling, caloric restriction, and even swim stress regulate GABA_A_ receptor subunit expression, at times with a lasting effect, suggesting that epigenetic influences may be as important in shaping the GABA_A_ receptor related differentiation and communication patterns [122,170, 202, 238].

### Developmental Aspects of GABA_A_ Receptor Signaling

**Fig. (2):**
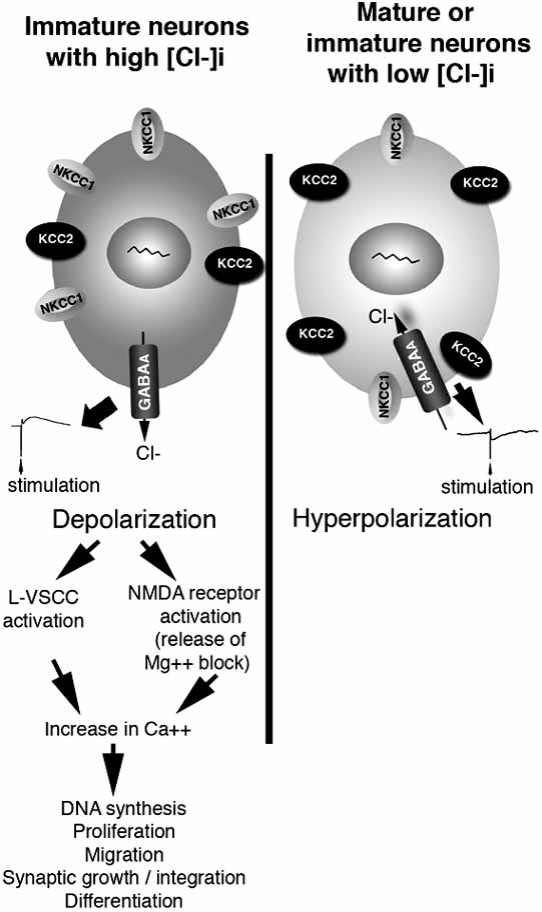
**The developmental switch in chloride cotransporter (CCC) expression drives the functional switch of GABA_A_ receptors from depolarizing to hyperpolarizing.**The developmental increase in KCC2 and, in certain tissues, the decrease in NKCC1 triggers the switch from depolarizing to hyperpolarizing GABA_A_ergic signaling [247, 261]. GABA-mediated depolarizations activate L-type voltage sensitive calcium channels(L-VSCC) and release the Mg++ block of NMDA receptors, increasing intracellular Ca++. This can activate calciumregulated signaling pathways, which are important in neuronal development, migration, proliferation, synaptogenesis and differentiation. The GABA-mediated activation of calcium signaling does not occur in neurons with hyperpolarizing GABA_A_ receptor responses.

GABA_A_ receptors, almost universally, depolarize very immature neurons [22, 23, 130, 177, 181, 198, 215, 235, 256, 275, 305]. The GABA_A_ergic depolarizations can activate voltage sensitive calcium channels, increase intracellular calcium and therefore activate calcium sensitive signaling cascades [23, 235, 256] (Fig. **2**). These are important for normal brain development, as they can control DNA synthesis, proliferation, migration, synaptic growth and integration and neuronal differentiation (Fig. **2**). For most of the studied neuronal types, there is a time in their maturation process, usually by the end of the first postnatal month in rats, when GABA_A_ergic signaling switches to hyperpolarizing [6, 15, 42, 79, 82, 97, 124, 127, 147, 149, 158, 167, 258, 261, 279, 295, 348, 349]. The ionic mechanisms implicated in this switch are related to the homeostatic regulation of chloride and bicarbonate ions, the main ions flowing through the chan-nel under normal conditions [4, 204, 220, 264, 303, 305].

As shown in Fig. (**3**), intracellular accumulation of chloride ions is favored when high levels of expression of sodium chloride cotransporters (NCCs), sodium potassium chloride cotransporters (NKCCs) or sodium-independent anion exchangers (i.e. AE3) occurs. In contrast, decrease in intracellular chloride occurs when potassium chloride cotransporters (KCCs), sodium dependent anion exchangers (NDAE) or chloride channel 2 (Clc2) are overexpressed. The ionic permeability of these proteins is graphically depicted in Fig. (**2**) and described in Table **1**. 

**Fig. (3) Schematic depiction of selected proteins involved in the regulation of Cl- homeostasis.**
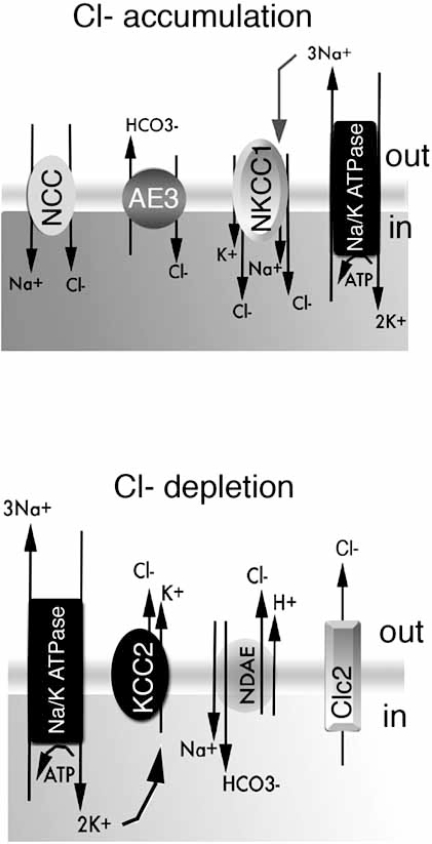
*Upper panel:* Cl- accumulation is effected by the presence of the *Na+/Cl-* *Cotransporter* NCC and *Na+/K+/Cl-* *Cotransporter* NKCCs, with main representative being NKCC1. Their function is dependent upon the supply of Na+ by the Na+/K+ ATPase. In contrast, *Anion Exchangers*, like AE3, favor Cl- accumulation in a sodium independent manner. *Lower panel*: Low intracellular Cl- concentration occurs as a result of *K+/Cl- Cotransporters*, such as KCC2, which export Cl- and K+. As a result, their function is also dependent upon K+ supply by Na+/K+ ATPases. The *Sodium Dependent Anion Exchangers* NDAE and *Chloride Channel 2* (Clc2) also decrease intracellular Cl-[69, 268].

The developmental change in chloride gradient across the open channel has long been implicated as a determining factor for the depolarizing and hyperpolarizing effects of GABA [4, 204, 220, 303, 305]. Very immature neurons have high intracellular chloride concentrations ([Cl-]_i_) that shift the equilibrium potential for Cl^-^(E_Cl-_) to values less negative than the resting membrane potential (Vm). As a result, opening of a GABA_A_ receptor channel leads to efflux of Cl -, which depolarizes the neuron in an attempt to reach E_Cl-_. Mature neurons have low [Cl-]_i_, E_Cl-_ values more negative than Vm, and hyperpolarizing responses to GABA_A_ receptor activation. The molecular characterization of chloride cotransport mechanisms (Table **1**, Fig. **3**) offered a first insight into the developmental regulation of GABA_A_ergic signaling. Cation chloride cotransporters (CCCs) mediate the electroneutral transport of Cl-along with either K+ (potassium chloride cotransporters, KCCs) or K+ and Na+ (sodium potassium chloride cotransporters, NKCCs) or Na+ only (sodium chloride cotransporters, NCCs) [60, 268]. Under normal conditions, KCCs extrude K+ / Cl-, decreasing intracellular Cl-, whereas NKCCs and NCCs import cations and Cl- into the cell, increasing intracellular Cl-. During the developmental period when GABA_A_ receptor signaling switches from depolarizing to hyperpolarizing in the hippocampus, the expression of key representatives of these families changes: NKCC1 decreases [247] whereas KCC2 increases [179, 261], with net result the decrease in intracellular Cl-. Furthermore, they are sufficient to trigger the switch as shown with *in vitro* or *in vivo* antisense inhibition [124, 261, 302,351], overexpression of KCC2 or NKCC1 [6, 39, 165] or pharmacological inhibitors of CCCs [333]. Similar age- and maturity-related changes in the expression of these cotransporters have been described in many neuronal structures [43, 79, 169, 179, 203, 281, 295, 316, 319]. Other factors that may contribute to the increased functionality of KCC2-mediated Cl-export in mature neurons is its more efficient localization at the plasma membrane and oligomerization [14, 26]. For NKCC1, a shift from a neuronal pattern to a glial-dominant pattern of expression has been described in the developing murine nervous system [123].

In normal humans, it is obviously difficult to identify the timing of the GABA_A_ receptor switch and related changes in CCCs. Using human brain tissue from patients deceased from non-neurological disorders, similar developmental increase in KCC2 over NKCC1 was identified in the cortex postnatally, suggesting that a similar gradient of GABA_A_ergic responses may occur [65]. Comorbid conditions and medical treatments, which are known to influence CCC expression and GABA_A_ receptor signaling, may, to an extent, have influenced the expression of these proteins. However, the resemblance of these patterns with the biology of the system in the experimental studies strongly supports the hypothesis that depolarizing GABA_A_ergic responses may indeed occur at least in prematurely born neonates. 

**Fig. (4):**
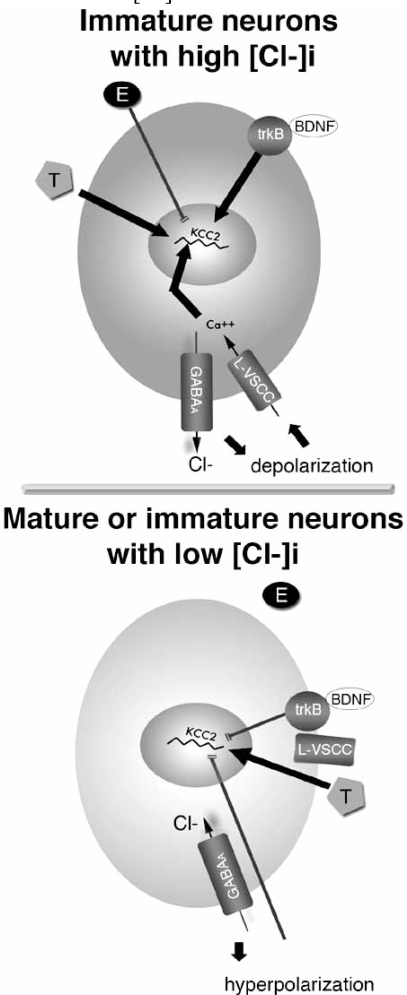
**Differential regulation of KCC2 in neurons with depolarizing or hyperpolarizing GABA_A_ergic signaling.**GABA_A_ receptor activation and BDNF increase KCC2 in immature neurons with depolarizing GABA_A_ ergic responses, but decrease it in neurons with hyperpolarizing GABA_A_ ergic signaling. Estradiol (E) downregulates KCC2 only in neurons with depolarizing GABA. Testosterone and its androgenic products (T) increase KCC2 in both conditions [3, 35, 81, 262, 263, 317].

Another level of complexity stems form recent findings that the maturation of GABA_A_ergic signaling and its regulators may occur earlier in females than in males. In the substantia nigra pars reticulata (SNR), the expression of KCC2 mRNA is always higher in female than in male GABAergic SNR neurons (infantile and juvenile period) [79]. This explains the earlier appearance of hyperpolarizing GABA_A_ergic responses in females than in males [79, 158]. Similarly, earlier appearance of hyperpolarizing GABA_A_ergic signaling was seen in dopaminergic neurons of the female rat substantia nigra pars compacta (SNC) [82]. As a result, during the sensitive developmental windows of divergent GABA_A_ergic signaling, physiological or pathological activation of these receptors may have distinct translational consequences in males and females. For instance, in male infantile (PN15) SN neurons, GABA_A_ergic depolarizations increase intracellular calcium, the expression of the phosphorylated form of the transcriptional factor CREB (cAMP responsive element binding protein), as well as the expression of calcium regulated mRNAs, such as KCC2 [79, 80, 82]. These do not happen in female PN15 SN neurons, in which GABA_A_ergic activation downregulates KCC2 mRNA [79]. Furthermore, GABA_A_ receptor signaling also interferes with estradiol signaling. Estradiol downregulates KCC2 mRNA only in neurons which are depolarized by GABA_A_ receptors [80] but not in neurons which are either hyperpolarized by them or in which GABA_A_ receptors have been blocked [80, 218] (Fig. **4**). These direct and indirect actions support that, in normal development, GABA_A_ receptors can act as broadcasters of sexually differentiating signals in the brain promoting its sexual differentiation [81]. 

In addition, the intracellular concentrations of Cl-and HCO3- are regulated by anion exchangers (AE). The sodium independent electroneutral AEs exchange HCO3- for extracellular Cl-, lowering intracellular pH and increasing Cl- [112, 300, 336]. *Sodium Dependent Anion* (Cl- / HCO3-) *Exchangers* (NDAE), also called sodium-dependent Cl-/HCO3- exchangers (NDCBE or NCBE) function in the opposite direction increasing intracellular pH and lowering intracellular Cl-[87, 92, 151, 287, 288, 315, 321]. The expression of NCBE precedes KCC2 in the embryonic mouse brain and, unlike KCC2, NCBE is expressed in the peripheral nervous system and epithelial non-neuronal tissues [125].

Finally, the hyperpolarization-activated chloride channel Clc2 has been implicated in maintaining low intracellular Cl- [41, 102,283, 292]. Clc2 mediated Cl- efflux is also enhanced by extracellular acidosis. Low expression of functional Clc2 has been reported in the rat neonatal hippocampus and has been correlated with the depolarizing actions of GABA_A_ receptors at this age [205]. Clc2 has also been proposed to be a key factor in maintaining low intracellular Cl- in adult dopaminergic neurons of the rat SNC [93]. 

GABA_A_ receptors are also permeable to HCO3-. As the equilibrium potential for HCO3-is approximately 50mV less negative than the resting potential, HCO3-flux is usually outward [294]. This renders the reversal potential of GABA_A_ergic inhibitory postsynaptic currents (E_GABA_) less negative than the E_Cl-_, although its contribution is much smaller compared to Cl- [5, 135, 136]. Upregulation of a cytosolic carbonic anhydrase (CAVII), which catalyzes the production of HCO_3_-from CO_2_, occurs in hippocampal pyramidal neurons around PN12, promoting the depolarizing GABA_A_ergic responses following high frequency repetitive stimulation [264].

An important distinction should be made though between the ability of GABA_A_ receptor activation to depolarize a neuron as opposed to excite a network into epileptiform discharges or seizure activity. GABA-mediated depolarizations can often reach the threshold for activation of voltage gated calcium channels, such as the L-type channels, or for release of Mg++ block of NMDA receptors (Fig. **2**). As a result these processes can increase intracellular calcium and activate calcium-sensitive signaling, with its known impact on brain development and differentiation. However, if the level of neuronal activation begins to exceed E_GABA_, which is very close to E_Cl-_, the open GABA_A_ receptors start to shunt excitation, by reversing Cl- flux, in an effort to maintain the neuronal potential close to E_GABA_ [293]. Undoubtedly, in conditions when E_GABA_ has shifted to significantly more positive values, even shunting inhibition can fail and this may explain reports of ictogenic properties of GABA [145, 209]. 

### Regulation of Chloride Cotransporters and GABA_A_ Receptor Signaling Switch

The functional importance of the switch of GABA_A_ergic signaling generated a lot of interest around regulatory factors underlying this process. Karadsheh and Delpire sequenced portions of the 5’ region upstream to KCC2 gene and identified a 21bp element with 80% similarity to the neuronal-restrictive silencing factor binding consensus sequence (NRSE) that may function as gene silencer [141]. Although this finding is in good alignment with the neuronal specificity of KCC2, further studies showed that KCC2 lacking this NRSE sequence remains neuronal specific; moreover, in the absence of this NRSE, KCC2 shows similar developmental increase as the normal gene [310].

A number of studies have also investigated the regulation of KCC2 and GABA_A_ergic switch by GABA signaling, showing that depolarizing GABA_A_ergic signaling is a positive drive for the developmental upregulation of KCC2 and switch of GABA_A_ receptors, mediating its effects through activation of voltage-sensitive calcium channels and activation of calcium signaling. Nevertheless it is not necessary, since in its absence the increase in KCC2 and GABA_A_ergic switch still occurs, albeit at a later timepoint. These have been shown *in vitro *using E18 dissociated rat hippocampal cultures [85] and rat E14 ventral midbrain neurons [308]. Further support has been provided with the effects of *in vivo* administration of GABA_A_ergic agonists and antagonists on KCC2 and GABA_A_ receptor switch in rat SNR [79] and turtle retina [167]. The sexually dimorphic features of the PN15 rat SNR have provided us with a convenient *in vivo* system to study KCC2 regulation in normal neurons with similar chronological age, which have either depolarizing (male) or hyperpolarizing (female) GABA_A_ergic signaling. The GABA_A_ergic agonist muscimol increases KCC2 mRNA in male neurons, *via* activation of voltage sensitive calcium channels and calcium signaling [79, 80]; in contrast, muscimol decreases KCC2 mRNA in female SNR neurons with hyperpolarizing GABA_A_ergic responses [79]. These indicate that the maturational state of a neuron, as it relates to the mode of GABA_A_ergic signaling, is critical in defining its reaction to stimuli that tend to disturb its GABA-related developmental pathway. On a separate note, Ludwig *et al.* did not observe any changes in KCC2 immunoreactivity in cultured PN0-1 hippocampal mouse neurons chronically treated with either picrotoxin and the sodium channel inhibitor tetrodotoxin (TTX) or combinations of TTX with glutamate receptor inhibitors, proposing that these are not necessary for the developmental increase in KCC2, in hippocampus [180]. Some of the differences in these results may be due to a combination of factors, such as the different maturational stages of the studied cells (embryonic rat *vs* postnatal mouse hippocampal), or the different combinations and doses of inhibitors. 

Another approach to dissect whether neuronal activation promotes the maturation of the GABA_A_ergic system has been through sensory deprivation or lesioning of the natural afferent stimulatory pathways to sensory nuclei. Unilateral or bilateral cochlear ablations prior to the onset of hearing, maintained KCC2 expression at low levels and prevented the developmental decrease in intracellular chloride – at least within the time frame of the study -, within the lateral superior olivary nucleus of the developing rat (~PN15) [280]. In turtle retina, dark rearing inhibited the developmental increase in KCC2 and prolonged the period of excitatory GABA_A_ergic responses [279]. 

Brain derived neurotrophic factor (BDNF) is a neurotrophic factor that has been implicated both in normal neuronal activity patterns, as well as in the mediation of long term effects of excessive and pathological patterns of neuronal excitability [3]. BDNF expression is high in the first 2-3 postnatal weeks and subsequently declines to adult levels (limbic system, rat ventral mesencephalon of voles) [173, 224]. BDNF exerts opposite effects on KCC2 expression, depending on the developmental stage of the target neuron. In developing neurons, BDNF increases KCC2 expression [3, 35], whereas in mature neurons BDNF decreases KCC2 and causes a positive shift of E_GABA _[262, 263, 317]. Rivera *et al.* identified trkB as the receptor involved in BDNF-mediated downregulation of KCC2 [263]. The PLCγ (phospholipase Cγ) signaling downregulates whereas Shc signaling upregulates KCC2 in their system [263]. The developmental and cell type specific expression of these signaling pathways may be therefore important in the developmental regulation of KCC2 by BDNF. 

CCCs are also functionally regulated by post-translational modifications. Tyrosine phosphorylation of KCC2 by insulin-like growth factor (IGF-1) and tyrosine kinases increase its activity [144]. Members of the serine-threonine kinase WNK (*With No lysine (K)*), SPAK (S*erine Proline Alanine lysine (K) rich*), and oxidative stress responsive (OSR) kinase families have drawn much focus in related research, showing that they are important, cooperatively or independently, in the activation of NKCC1, NKCC2, NCCs and de-activation of KCCs [77, 86, 134] [259] [57]. These interactions are important for the volume-regulation of CCC activity. Activation of protein kinase A pathway (PKA) pathway, through its effects on protein phosphatases, has been implicated in the activation of KCC2 following high frequency stimulation of PN2-3 rat neurons at the deep cerebellar nuclei [234]. Platelet-derived growth factor (PDGF) activates KCC2 *via* the PI 3-K / PP-1 pathway (phosphoinositide 3-kinase / protein phosphatase-1) [346]. Although certain systems may be more sensitive to modulators of the activity of similar kinases [148], it is not yet known how they contribute to the developmental changes in CCC activity and Cl- regulation.

Hormonal regulation of CCC function is also important during development, given the ongoing neuroendocrine changes occurring at this period, which are important for brain development. We have studied the regulation of KCC2 by sex hormones in PN15 SNR, using *in vivo* injections. Testosterone and its androgenic derivative dihydrotestosterone both increased KCC2 mRNA expression acutely and this effect was sustained after repetitive doses. This androgenic effect was observed both in male and female SNR, suggesting that it can occur regardless of the direction of GABA_A_ergic signaling [80]. Interestingly, 17β-estradiol was effective in decreasing KCC2 mRNA only in SNR neurons with depolarizing GABA_A_ergic signaling, suggesting an interaction of the two pathways [80]. In accordance with these findings, estrogens failed to regulate KCC2 expression in the pyramidal region of the hippocampus of adult ovariectomized females, which likely have mature GABA_A_ergic responses [218]. 

### Implications for Normal Development and Physiology

In embryonic and immature neurons, GABA has neurotrophic properties: it regulates the proliferation, migration and differentiation of neurons, dendritogenesis and synaptogenesis, increases the number of neurotubules, rough endoplasmic reticulum, Golgi apparatus, synaptic vesicles [20, 23, 39, 105, 291]. As the functional recruitment and requirements of each neuronal structure during development changes with different tempos, it is not surprising that the maturation of the GABA_A_ergic signaling pathway occurs at different timepoints for each cell type. The sensitive regulation of GABA_A_ergic signaling by neuronal activity, patterns of sensory input, epigenetic factors, hormonal influences, interaction with other signaling pathways ensures that brain development will occur in a patterned but also time-, context-, sex-, and experience-driven fashion. This asynchronous maturation may at times be important for structured communication between different cell types [320] or generation of specific activity patterns [279]. On the other hand, it also renders it very vulnerable to dysfunction in case of pathological influences, as will be described in the subsequent sections.

## GABA_A_ RECEPTORS IN SEIZURES AND EPILEPSY

GABA_A_ ergic drugs are the mainstay of treatments to suppress seizures [118, 199, 249, 266, 323]. They are primary or secondary targets of many of the available anticonvulsants [199]. These include drugs enhancing GABA_A_ receptor action through a direct interaction with the receptor (benzodiazepines, barbiturates, propofol, stiripentol, topiramate, carbamazepine, phenytoin, felbamate) or indirectly by increasing the available GABA (tiagabine, vigabatrine, gabapentin, valproate) [51, 90, 156, 199, 251]. Furthermore, anticonvulsants can reduce the depolarizing effects of GABA_A_ receptors by inhibiting carbonic anhydrase (topiramate, zonisamide, acetazolamide) [58, 63, 192, 223, 257]. 

GABA_A_ receptors may influence the susceptibility to seizures. A variety of epileptic or seizure syndromes have been linked to genetic mutations of GABA_A_ receptors, which compromise their function (Table **1**). Seizures are most prevalent during the neonatal and infantile period, a time when the brain has not fully matured [103, 104, 212]. Although it is difficult to extrapolate experimental data to humans, this is the time when expression, efficacy, subcellular localization of GABA_A _receptors, and functional maturation of GABA-driven subcortical seizure-controlling networks have not been fully optimized [314]. For example, GABA_A_ergic activation of the anterior SNR exerts proconvulsant effects in PN15 rats but anticonvulsant effects in PN30 male rats [313]. Moreover, shunting inhibition, due to the depolarizing E_GABA_ is expected to be less efficient. This was nicely demonstrated both *in vitro* and *in vivo* by Dzhala *et al.* who showed that bumetanide, an NKCC1 inhibitor, can suppress ictal activity in very young rats [65]. Its efficacy dropped though in older ages, probably due to the decreased expression of NKCC1. In older preparations or subjects, anticonvulsant efficacy has been demonstrated for compounds potentially inhibiting other age-appropriate mechanisms mediating GABA_A_-depolarizations. These include thiazides and carbonic anhydrase inhibitors (acetazolamide) [114, 189, 274]. Furosemide, a loop diuretic preferentially inhibiting KCC2 over NKCC1, has been shown to have anticonvulsant activity, but its effect has been linked to a decrease in neuronal synchronization and cell volume regulation [116]. 

By far the most common type in patients with intractable epilepsy is temporal lobe epilepsy (TLE) [12, 30, 66, 78]. In most cases, TLE has not been linked to genetic factors. TLE patients commonly have a history of an initial precipitating event (IPI), including prolonged neonatal seizures [193]. As a result, intense research is undergoing to reveal how changes in GABA_A_ergic signaling interfere with the acquired mechanisms of ictogenesis, epileptogenesis, and medical refractoriness. In human specimens from resected temporal lobes of patients with TLE and hippocampal sclerosis, the associated neuronal loss results in decreased cell counts of GABA_A_ receptor immunoreactive cells in the vulnerable regions (CA1, CA3, hilus) [178]. The surviving neurons and interneurons show changes in morphology, expression and subcellular distribution of GABA_A_ receptor subunits that partially correspond to patterns seen in younger age groups, based in the experimental studies. Specifically, these changes include increase in α2, α1, β2, β3, γ2 subunit expression in the somata and apical dendrites but reduction in basal dendrites, decreased α1 expression in sectors CA1, CA2, and CA3, decrease of α1 and increase of α2 in CA2 [178]. Pharmacologically, these studies may be interpreted as suggesting that the epileptic state may be associated with less sensitivity to GABA_A_ergic drugs, specifically to benzodiazepines, at least in certain hippocampal neurons. Using flumazenil (benzodiazepine antagonist) PET study, Chugani *et al.* studied a cohort of patients with epilepsy (2-17 years old) and found an age-related decrease in flumazenil volume of distribution; this change occurred earlier in the subcortical regions [40]. From the experimental models, it is obvious that some of these changes may occur after prolonged seizures and may, at least in certain cases, be long lasting or permanent (Fig. **5**). Interestingly, the effects of prolonged seizures on GABA_A_ receptor expression and function are different in younger rats (Fig. **5**), an observation that may partially explain the different outcomes of SE in very young *vs* older subjects. Despite its higher susceptibility to seizures, the immature brain is relatively more resilient to acute injury, epileptogenesis and long term cognitive dysfunction than the mature brain [78, 212]. 

Beyond its role in suppressing seizures, several groups have proposed that GABA_A_ergic signaling, under certain conditions, contributes to the appearance of interictal epileptic discharges by increasing neuronal synchronization [13, 153, 154]. An important study that re-focused the interest upon the role of depolarizing GABA in human mesial TLE was published in 2002 [44]. The authors showed that interictal-like activity detected *in vitro* from the subiculum of patients who underwent resective surgery for mesial TLE was blocked by either GABA_A_ or glutamate receptor antagonists. This was associated with a positive shift in E_GABA_ of the pyramidal subicular neurons. Palma *et al.* independently concluded that cell membranes from human epileptic tissue, when injected into Xenopus oocytes, elicited depolarizing GABA_A_ergic currents, which was linked to upregulation of NKCC1 and downregulation of KCC2 [237]. Bertelli *et al.* have recently reported decreased levels of a Clc2 isoform in epileptic temporal lobes [25]. Furthermore, in patients with cortical dysplasias and intractable epilepsy, high expression of NKCC1 and abnormal subcellular distribution of KCC2 has been shown by 2 groups [9, 216]. Similar observations have been obtained in experimental models of seizures. Hippocampal kindling of adult mice decreased KCC2 expression in the hippocampus, through activation of the BDNF / trkB pathway [262]. Amygdalar kindling of adult rats increased NKCC1 in the piriform cortex [231] and dentate gyrus [232]; decreased KCC1 and Clc2 in the dentate gyrus and Clc2 in the CA1 pyramidal region of the hippocampus, but had no effect on KCC2 [232]. In their *in vitro* model of mirror epileptogenic focus, Khalilov *et al.* describe that seizure propagation to a drug-naïve hippocampus aberrantly switches GABA_A_ergic signaling back to its immature depolarizing mode and this is important for the generation of ictal patterns [145, 146]. It should be noted that all these studies pertain to neurons, which at the time of seizures had mature-type, hyperpolarizing GABA_A_ergic responses. Given the divergent patterns of regulation of KCC2 in neurons with depolarizing *vs* hyperpolarizing GABA_A_ergic signaling (Fig. **2**), can these observations be extended to neonatal and pediatric epilepsies, when the brain is still immature? 

Isaeva *et al.* induced repetitive but brief flurothyl-induced seizures (not SE) in neonatal rats [129]. In their model, they failed to see any significant effect of neonatal seizures on the timing of GABA_A_ergic switch in the CA3 region of the hippocampus, albeit the amplitude of IPSCs was reduced. More severe and prolonged seizures induced as 3 neonatal episodes of kainic acid-induced SE, increase KCC2 mRNA expression in the CA3 pyramidal region of the male rat hippocampus [83]. A possible explanation is that activation of BDNF and GABA_A_ergic signaling during neonatal SE may actually upregulate KCC2 (Fig. **2**). Both these studies support that the seizure-induced re-appearance of depolarizing GABA_A_ergic responses observed in mature neurons is unlikely to occur in the neonatal brain with immature GABA_A_ergic signaling. If indeed the epileptic state is linked with aberrant maintenance of depolarizing GABA_A_ergic signaling, these findings may explain why neonatal rats are relatively resistant to the development of epilepsy following neonatal SE. Further studies are underway to fully characterize the effects of neonatal seizures on chloride homeostasis and GABA_A_ergic switch and determine how these may contribute to the different outcome of neonatal seizures on brain development [78, 117]. 

Finally, a number of conditions that increase risk of subsequent epilepsy induce aberrant switch of hyperpolarizing to depolarizing GABA_A_ergic signaling in mature neurons. These include hypoxia [84], axonal injury [217]. Interestingly, hypoxia in immature neurons decreases intracellular Cl- [334].

## CONCLUSIONS 

There is undoubtedly wide region, sex, age, species, experience-driven diversity in GABA_A_ergic signaling. These differences may seem subtle, often identified only with sensitive pharmacological, electrophysiological or immunological tools, suggesting that its complex components serve as fail-safe mechanisms to preserve an important homeostatic mechanism. Their temporal evolution serves sex-, cell type-, and age-appropriate functions: neurotrophic and morphogenetic early in development; activity-driven plasticity at the time when environmental cues make their maximal imprint on the structural and functional organization of the brain; and finally inhibitory and neuromodulatory when the mature brain needs to homeostatically preserve its learned patterns of activity. A key feature of the immature type function of GABA_A_ receptors is the depolarizing signaling, attributed to the inability of young neurons to maintain low intracellular chloride. This is critical for age- and sex-appropriate brain development and differentiation. Of equal importance is to keep in mind that the regulation of GABA_A_ergic switch is different in neurons with depolarizing *vs* hyperpolarizing GABA_A_ergic signaling. In mature neurons, recurrent and prolonged seizures may trigger a pathological reemergence of immature features of GABA_A_ receptors, which compromises the efficacy of GABA-mediated inhibition. In immature neurons with depolarizing GABA_A_ergic signaling, the physiological and pathological regulation of this system is completely different, possibly contributing to the different outcomes of early life seizures. Moreover, since disturbing the timing of GABA_A_erging switch can potentially have long lasting effects on brain development and differentiation, it becomes increasingly more urgent to design sex- and age-specific pharmacological interventions adapted for the maturational stage of the targeted brain region, so as to limit side effects. Of particular relevance is the further characterization of the long-term effects on naïve fetuses exposed *in utero* to maternal use of drugs acting on this system.

## Figures and Tables

**Fig. (5)Schematic depiction of the timeline of changes in GABAA receptor subunit mRNA expression in the hippocampus, in rodent models of temporal lobe seizures and epilepsy.**
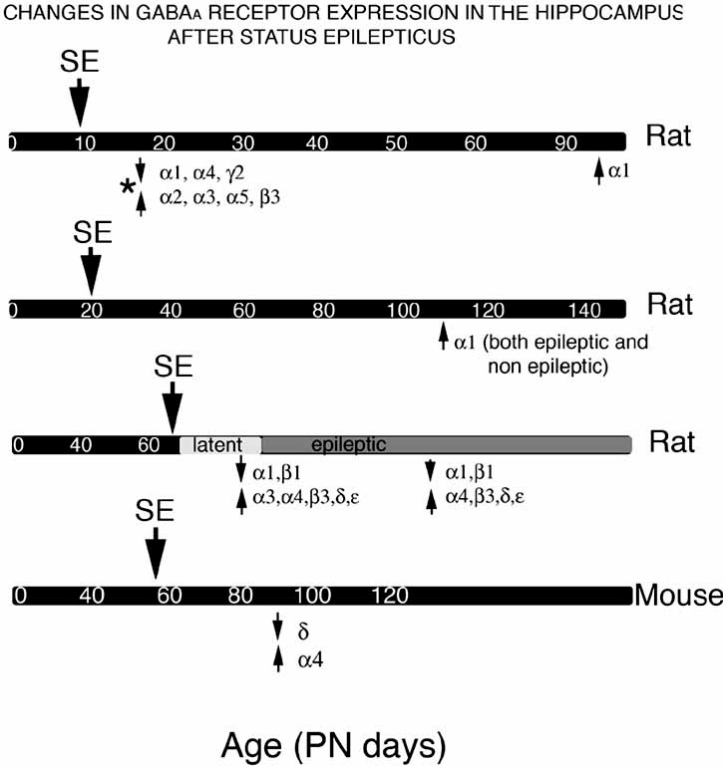
The effects of SE change according to age of induction, model, and species. In most cases, the results stem from the lithium-pilocarpine or pilocarpine SE model, except for the results marked with an asterisk, which were described after kainic acid SE. Adulthood starts at PN60. The time scale used for the effects of SE in adult rats is approximate and is meant to reflect changes during the latent phase of epileptogene-sis, prior to the onset of spontaneous seizures, and during the epileptic phase, ie after the occurrence of 2 spontaneous seizures. The diagrams are based on a review of the pertinent literature [33, 73, 163, 254, 244, 345].

**Table 1.** Selected Proteins Involved in Cl- Transport [106, 241, 268].

Protein	Ion Permeability	Features	Inhibitors	Disease Linkage	References
**Cation chloride cotransporters**					
Potassium Chloride Cotransporters (KCCs)					[57, 77, 86, 134, 259]
KCC1 (SLC12A4)	Efflux of K+, Cl-	Ubiquitous	Inhibited by: Furosemide > bumetanide, DIOA, ATP, Hypertonic media, Disulfonic acid stilbene High extracellular K+, WNK, SPAK Activated by: Hypotonic media;N-ethylmaleimide;PDGF (KCC2);PKA (KCC2)	N/A	[86, 88, 119, 134, 227, 242, 299, 347, 350]
KCC2 (SLC12A5)		Neuronal specific		N/A	[77, 86, 134, 234, 239, 263, 290, 346]
KCC3 (SLC12A6)		Widespread expression: heart, kidney, neurons, epithelia, red blood cells, muscle, placenta		ACCPN ; variants with bipolar disease	[86, 115, 121, 200, 201, 213, 252]
KCC4 (SLC12A7)		Widespread expression; weak in brain		N/A	[86, 169, 213, 312]
Sodium potassium chloride cotransporters (NKCCs)					
NKCC1 (SLC12A2; BSC2)	Influx of Na+, K+, 2Cl-	Ubiquitous	Inhibited by: Bumetanide > Furosemide PP-1 Activated by: ATP, Hypertonic media, Calyculin A, Low Cl WNK, SPAK (NKCC1)	N/A	[49, 59, 77, 134, 210, 240, 246, 247]
NKCC2 (SLC12A1; BSC1)		Kidney		Bartter’s syndrome type I	[76, 126, 188, 259, 284]
Sodium chloride cotransporters (NCCs)						
Sodium chloride co-transporter (SLC12A3; NCC)	Influx of Na+, Cl-	Kidney	Inhibited by: Thiazides Activated by: WNK	Gitelman’s syndrome	[259]
**Chloride channels**						
Cl- channel 2 (Clcn2 or Clc2)	Efflux of Cl-	Brain (neurons), heart, pancreas, lung, liver, fibroblasts, epithelial	Inhibited by: PP-1 Activated by: Hyperpolarization, cell swelling, acidic pH, hypo-osmotic shock, arachidonic acid, omeprazole, p34(cdc2)/cyclin B; PKA	Idiopathic generalized epilepsy	[25, 47, 52, 72, 75, 102, 222, 286, 306]
**Selected HCO3- transporters**						
Na+-dependent anion exchanger NDAE (SLC4A8; NDCBE)	Influx Na+, HCO3-; Efflux H+, Cl-	Brain, testis, kidney, ovary	Inhibited by: DIDS Activated by: ATP requirement (squid)	N/A	[92, 267,]
Na+-independent anion exchanger AE3 (SLC4A3)	Influx Cl-; Efflux HCO3-	Brain, retina, heart, smooth muscle, epithelia	Activated by: Increased intracellular pH	Idiopathic generalized epilepsy	[267, 273]

**Abbreviations:** ACCPN: Agenesis of Corpus Callosum with Peripheral Neuropathy; DIDS: 4,4’-diisothiocyano-2,2’-stilbene disulphonic acid; PP-1: protein phosphatase 1; PKA: protein kinase A; PDGF: platelet derived growth factor; WNK: *With No lysine (K)*; SPAK: *Serine Proline Alanine lysine (K) rich*; OSR: oxidative stress responsive kinase; DIOA: dihydronindenyloxy alkanoic acid.

**Table 2.** Epileptic and Seizure Syndromes Associated with Abnormalities in GABA_A_ergic Signaling

Gene Defect	Epileptic Syndrome	Proposed Dysfunction	Reference
**GABA_A_ receptor subunits**			
α 1 (GABRA1)	Autosomal dominant juvenile myoclonic epilepsy (ADJME)	Low amplitude GABA currents; reduced surface expression; increased GABA EC50	[45, 184]
β3 (GABRB3)	Childhood absence epilepsy		[71]
Deletion in 15q11-q13 (includes β 3,α 5, γ 3)	Angelman syndrome		[159, 301]
γ 2 (GABRG2)	Autosomal dominant epilepsy with febrile seizures plus (ADEFS+)	Impaired Cl- influx or potentiation by endozepine	[19]
	Febrile seizures + childhood absence epilepsy	Impaired sensitivity to benzodiazepines; accumulation of desensitized receptors; endoplasmic reticulum retention; temperature-sensitive trafficking defect	[70, 137, 139, 184, 191, 270, 318]
	ADEFS+ including a patient with severe myoclonic epilepsy of infancy (SMEI)	endoplasmic reticulum retention	[98]
	Febrile seizures	Increased fast phase desensitization, reduced sensitivity to diazepam	[10, 38]
δ (GABRD	ADEFS+	Low amplitude GABA currents	[62]
	ADEFS+, idiopathic generalized epilepsies (IGE), febrile seizures, but also controls	Low amplitude GABA currents	[62]
	ADJME	Low amplitude GABA currents	[62]
**Proteins involved in Cl- regulation**			
AE3 (SLC4A3)	IGE	Abnormal Cl- homeostasis ?	[273]
Clc2 (Clcn2)	IGE	Lower transmembrane Cl- gradient, altered voltage-dependent gating	[52, 102, 297]
